# Burnt Out and Dropping Out: A Comparison of the Experiences of Autistic and Non-autistic Students During the COVID-19 Pandemic

**DOI:** 10.3389/fpsyg.2021.792945

**Published:** 2022-01-03

**Authors:** Eilidh Cage, Ellie McManemy

**Affiliations:** Department of Psychology, Faculty of Natural Sciences, University of Stirling, Stirling, United Kingdom

**Keywords:** autism, burnout, mental well-being, university dropout, higher education, COVID-19

## Abstract

Autistic students are more likely to drop out of university, while facing both challenges and opportunities within university environments. This study compared the experiences of autistic and non-autistic current United Kingdom students, in terms of thoughts about dropping out, burnout, mental health and coping, during the COVID-19 pandemic. Burnout was of particular interest as this is a relatively unexamined phenomenon for autistic students. Seventy autistic and 315 non-autistic students, completed a mixed methods questionnaire with standardized measures of burnout (personal and academic), mental health (depression, stress, and anxiety), and coping styles (adaptive and maladaptive). We also included qualitative questions about dropping out and COVID-19 experiences. We found autistic participants experienced higher rates of burnout and mental health symptoms and were more likely to have thought about dropping out. Reasons given for thinking about dropping out, for both groups, focused on poor mental well-being, doubts about university, and academic challenges. For autistic participants, further analyses did not identify specific predictors of thinking about dropping out, but for non-autistic participants, this was predicted by maladaptive coping styles and academic burnout. Academic and personal burnout predicted one another for autistic students, and age, maladaptive coping, autistic characteristics, stress, and anxiety additionally predicted burnout for non-autistic students. Similarities in experiences during the pandemic were noted, with both groups experiencing negative social implications, difficulties adjusting to emergency online learning, and poorer psychological well-being. Moving forward from COVID-19, universities must find ways to enhance both academic and social support, to enable equal opportunity within Higher Education for autistic students.

## Introduction

Autistic individuals^1^ experience differences and difficulties within social interactions and social communication (particularly when interacting with non-autistic individuals; Crompton et al., 2020), differences in sensory processing (Clince et al., 2016), passionate and focused interests (Grove et al., 2018), stimming (self-stimulating) behaviors (Kapp et al., 2019), and preferences for routine or familiarity (Grove et al., 2018). Autistic people can have a wide range of support needs, and each autistic person will experience a different constellation of strengths and challenges. Many autistic people are now deciding to pursue Higher Education. For example, in the United Kingdom in 2019/20, 14,360 students disclosed that they were autistic, compared to 6,845 in 2014/15 (HESA, 2021). However, the actual number of autistic students at university could be much higher, as many choose not to disclose (Knott and Taylor, 2014) or have experienced barriers to diagnosis (Huang et al., 2020). One additional concern is that many autistic students appear to be more likely to drop out of university than their non-autistic peers (Newman et al., 2011; Cage et al., 2020). It is therefore imperative we understand why autistic students might be more likely to drop out, and generally better understand how to improve autistic students’ experiences at university.

However, previous research on university completion for autistic students is limited. Cage et al. (2020) conducted a survey of 230 autistic people (mostly from the United Kingdom), of whom 45 had not completed their studies, 151 had graduated and 34 had graduated after several attempts. Those who did not complete reported a poorer academic experience, found the transition to university more challenging and felt less like they fitted in to their university. Cage and Howes (2020) carried out semi-structured interviews with 14 autistic people who had dropped out of university in the United Kingdom, identifying systemic, societal issues that related to the participants’ decision to drop out, as well as challenges within the university environment, such as feelings of culture shock, disengagement with their studies, and experiencing a lack of proactive support. Anderson et al. (2020) interviewed 11 autistic students from Australia and New Zealand, of whom three had not completed their degree. The reasons they had withdrawn related to poor mental and physical health, sensory challenges, low motivation for the degree subject, and lack of support. In a study of current United Kingdom autistic students, Gurbuz et al. (2019) reported that more autistic students had considered withdrawing (56%) than non-autistic students (15.3%), but did not explore their reasons for feeling this way in depth. Adding to the limited literature on this topic is important, to better understand the mechanisms underlying dropout for autistic students and to ensure that autistic students are experiencing an equality of opportunity when it comes to Higher Education.

Until recently, there have been few direct comparisons between autistic and non-autistic students in the autism research literature. Comparing may help us better understand aspects of the university environment which particularly affect the experiences of autistic students over their non-autistic peers, and to identify mechanisms contributing to dropping out specifically for these students. Gurbuz et al. (2019) used a mixed methods online survey to compare the experiences of 26 autistic and 158 non-autistic students from the United Kingdom. Autistic students self-reported higher mental health difficulties and more challenges with social aspects of university, which Gurbuz et al. (2019) suggested linked to dropping out intentions. Other studies comparing autistic and non-autistic students have not considered dropping out. For example, Lei and Russell (2021) interviewed 18 autistic and 18 non-autistic students from the United Kingdom about perceptions of their self-determination (their ability to determine their own future and experiences) at university, noting that while there were commonalities, autistic students discussed autistic-specific strengths and more difficulties with transitions. Gillespie-Lynch et al. (2020) compared the writing skills of 25 autistic and 25 non-autistic students in the United States, finding autistic students expressed more writing skill and quality, higher nonverbal intelligence, and more perfectionist attitudes toward writing. These studies indicate there are unique strengths and challenges experienced by autistic students and comparing could help us to identify autistic-specific support versus support that would benefit students more broadly.

It is worth considering autistic students’ experiences in relation to the higher prevalence of mental health difficulties in the broader autistic population (Lai et al., 2019), which may contribute to the risk of dropping out for autistic students. For example, Eaves and Ho (2008) found that 77% of young autistic adults had co-occurring mental health conditions, with depression one of most common mental health conditions for autistic people (Gillott and Standen, 2007; Hollocks et al., 2019). Studies have also suggested that anxiety, including social anxiety, is significantly higher in the autistic population than the non-autistic population (Maddox and White, 2015). It is perhaps not unforeseen, then, that autistic students also report experiencing more mental health difficulties (Gurbuz et al., 2019), although there is little research directly measuring and comparing mental health symptoms experienced by autistic and non-autistic students or examining poor mental health as a predictor of dropout intentions.

Indeed, mental health difficulties in the student population (irrespective of whether someone is autistic) have been a concern for several years (Barkham et al., 2019), with the university period viewed as a time of distress (Bewick et al., 2010). Increasing numbers of students report mental health conditions: in the United Kingdom, in 2014/15, 33,500 students disclosed a mental health condition to their university, and in 2019/20 this was 96,490 (HESA, 2021). However, many students can find it difficult to disclose (Woodhead et al., 2020), suggesting numbers could be significantly higher than recorded statistics indicate. Given the high prevalence of mental health difficulties generally among the student population, research aiming to understand this prevalence plus appreciating the intersection between mental health and being autistic could tell us more about what could happen to improve university experiences for these students and to prevent dropout.

One aspect of mental well-being that has not been considered within the university context nor in relation to dropping out for autistic students, to the best of our knowledge, is burnout. Burnout is typically described as a state induced by stress, feeling mentally and physically exhausted, depersonalized, and unaccomplished (Pines and Maslach, 1978). Initially a phenomenon studied within workplace contexts, it has also been considered in relation to student experiences (Fernández-Castillo, 2021). For example, in non-autistic students, Zhang et al. (2007) suggest that burnout is prominent among students due to the high demands of balancing university with other life stressors. In a study of 7,757 university students, Portoghese et al. (2018) found that 34.2% were burned out, and a further 51% were “overextended,” meaning that they displayed moderately high levels of exhaustion. Prior research has also indicated that for non-autistic students, burnout can predict dropout intentions (Dyrbye et al., 2010; Marôco et al., 2020; Mostert and Pienaar, 2020) and has been related to suicidal ideation (Dyrbye et al., 2008).

Researchers generally poorly understand the topic of burnout for autistic people, particularly whether there is a specific phenomenon of “autistic burnout.” Using thematic analysis, Raymaker et al. (2020) explored how autistic people defined autistic burnout. They characterized autistic burnout as consisting of long-term chronic exhaustion, being less tolerant of stimuli (e.g., sensory stimuli), and loss of skills (e.g., being able to remember things, socialize or regulate emotions). They described autistic burnout as happening due to intense life stressors, inadequate support, and when expectations exceeded abilities. Using a Grounded Delphi study with 23 autistic adults, Higgins et al. (2021) developed a conceptual framework for autistic burnout. Their findings somewhat corroborate Raymaker et al. (2020), with autistic burnout defined as consisting of chronic exhaustion, reduction in daily living skills, interpersonal withdrawal, and increased difficulties with executive functions. Higgins et al. (2021) acknowledge some of the similarities with standard definitions of burnout but argue for distinctions around the cognitive effects and unique drivers of autistic burnout (such as those in relation to sensory sensitivities).

More work is needed to fully understand autistic burnout, particularly within the Higher Education context, and whether it contributes to thoughts about dropping out. In our study, as we compared the experiences of autistic and non-autistic students, a standardized measure of burnout was used rather than an autistic-specific measure [which has only recently been in development (Raymaker et al., 2020)]. We considered both academic burnout (i.e., burnout specifically related to university and academic demands) and personal burnout [i.e., burnout outside of university demands – how physically and psychologically exhausted someone is in general (Kristensen et al., 2005)]. By comparing experiences, this helps us further understand whether autistic burnout should be conceptualized as distinct, while also appreciating that an autistic student could also experience academic burnout in addition to autistic burnout.

Given the discussed prevalence of mental health challenges and risk of burnout within university contexts, for both autistic and non-autistic students, we should also consider how students cope with the stress they experience at university. Coping refers to attempts taken to reduce or prevent stress, harm, or threat (Carver and Connor-Smith, 2010). University can be a stressful environment, where students need to use coping strategies to get by (Böke et al., 2019). Some coping strategies may be considered more adaptive, whereby the means of coping supports positive adaptation to stress (such as seeking social support), or maladaptive, where ultimately unhelpful or potentially harmful strategies are used (such as substance abuse; Brougham et al., 2009; Sirois and Kitner, 2015). Research with students indicates that coping styles can play a role in well-being, for example by maladaptive strategies contributing to poorer well-being (Tran and Lumley, 2019), while more positive well-being is related to greater use of adaptive strategies (Freire et al., 2016). However, there is a significant lack of research exploring the coping strategies of autistic students, with coping only mentioned within a few qualitative studies (Van Hees et al., 2015; Cai and Richdale, 2016). We also do not know how coping strategies may relate to dropping out for autistic students.

Finally, we must consider all the discussed points within the context of a global pandemic, and its associated restrictions, which may have exacerbated challenges for both autistic and non-autistic students. The data for this study were collected during the COVID-19 pandemic, which changed Higher Education in many ways – such as universities shifting rapidly to online teaching and assessments, the loss of social events and connections, and increasing concerns over the job prospects of students (De Man et al., 2021). Research on students during the pandemic indicated high rates of depression and anxiety (Cao et al., 2020; Kaparounaki et al., 2020; Odriozola-González et al., 2020; Savage et al., 2020; Birmingham et al., 2021), with high academic stress, institutional dissatisfaction and fear of catching COVID-19 associated with increased depression (De Man et al., 2021). A study of over 30,000 students from 32 different countries noted emotions around frustration, boredom, and anxiety, with many worried about their future studies and careers, with some inequalities in relation to different socio-demographic characteristics (Aristovnik et al., 2020). Students also experienced increased loneliness during the pandemic lockdowns (Bu et al., 2020).

Research on autistic people’s experiences during the pandemic has generally not had a student focus. However, one study in the United States surveyed 76 autistic students (using non-standardized measures), noting particular anxiety around catching or spreading COVID-19, and managing their studies online (Monahan et al., 2021). In qualitative answers, these students also reported concerns over keeping up academically, getting support, and being able to attend and participate in online classes. Other studies have included autistic students in combination with other disabled students: Gin et al. (2021) categorized autistic students alongside students with learning disabilities (although autism is not a learning disability), and found that overall for disabled students, many could not access accommodations when learning moved online. Soria et al. (2020) grouped autistic students with other students with “neurodevelopmental or cognitive disabilities” (e.g., ADHD), noting that these students were more likely to experience financial difficulties, low feelings of belonging, and felt a lack of support. Soria et al. (2020) also reported that disabled students experienced higher symptoms of depression and anxiety than non-disabled students during the pandemic.

Other studies without a student focus provide some additional insight into the experiences of autistic people generally during the pandemic. Adams et al. (2021) conducted an online survey of 275 autistic adults in the United States 10 weeks into the pandemic, with data available on mental health symptoms pre- and during the pandemic. They found no significant change in mental health symptoms, but higher COVID-related distress was related to increased depression and anxiety during the pandemic. Another United States study found that autistic females, those with a prior mental health diagnosis, and those who knew someone with COVID-19, reported higher levels of psychological distress (Bal et al., 2021). In a sample of 1,044 autistic adults from Belgium, Netherlands, and the United Kingdom, Oomen et al. (2021) found increased depression and anxiety for autistic adults during the pandemic, as well as many missing social contact. Pellicano et al. (2021) interviewed autistic adults, autistic young people, and parents (autistic and non-autistic), mostly from Australia, noting that although lockdowns brought fewer social pressures and reduced masking, many reported a negative impact of not being able to connect with other people, with mental health deteriorating. Together, these studies show the overall negative impact of the pandemic for autistic people, although we do not know much about autistic students’ experiences.

Overall, the current exploratory study aimed to examine and compare autistic and non-autistic students’ experiences in relation to dropout, burnout, mental health, coping, and the COVID-19 pandemic, using mixed methods. The research questions were:

1. Are there differences in considering dropping out, burnout, coping styles, and mental health between autistic and non-autistic students?
2. Do these variables (poor mental health, greater burnout, and maladaptive coping styles) predict whether autistic and non-autistic students consider dropping out or not?
3. Do these variables predict burnout for autistic and non-autistic students?
4. How has the COVID-19 pandemic affected autistic and non-autistic students?

## Materials and Methods

### Participants

In total, 385 participants from the United Kingdom took part, of which 315 were non-autistic and 70 were autistic. Most participants were undergraduates (88.1%) with 11.6% postgraduate students. 178 were studying a STEM subject, 184 Arts and Humanities, and 23 preferred not to say. We recruited participants online using social media platforms (e.g., Facebook, Reddit, Instagram, and Twitter) by posting the questionnaire link along with a study advert. We also used the University of Stirling’s research participation system, which encourages students to participate in research projects in exchange for module credit. Autism-specific groups on social media were also used for recruitment of autistic students. Recruitment took place in November and early December 2020. This coincided with a period of tiered restrictions in Scotland (where local areas had different levels of restrictions imposed), and the vaccine roll-out beginning in December 2020.

For autistic participants, both those with formal diagnoses and those who suspected they were autistic, but were not formally diagnosed, were included, due to barriers to diagnosis (Huang et al., 2020). 32 participants were formally diagnosed and 38 were self-identifying. People not fitting the stereotypical view of “autism” are less likely to be diagnosed, particularly females (Lockwood Estrin et al., 2021), and in our study 76% (*n* = 29) of those self-identifying as autistic were female. All of the autistic participants (formally diagnosed and self-identifying) scored above the cut-off score of 14 on the RAADS-14, a screening tool for autistic characteristics. Due to the COVID-19 pandemic, we were unable to independently verify whether self-identifying autistic participants would meet diagnostic criteria using in-person assessments. Due to the exploratory nature of this study, and the importance of not gatekeeping or invalidating the experiences of those who have self-identified (Lewis, 2016a,b), we retained these participants in the sample. Autistic participants were significantly older than non-autistic participants [autistic mean = 24.16 (*SD* = 6.84); non-autistic mean = 21.35 (*SD* = 3.51), *t*(379) = −4.93, *p* < 0.001]. Other demographic characteristics are noted in Table 1, with notably most students being female, Scottish, and White, for both groups.

**Table 1:** Demographic information and participant details for autistic and non-autistic students.

	Non-autistic	Autistic
Gender		
Male	14.3% (*n* = 45)	17.1% (*n* = 12)
Female	85.4% (*n* = 269)	71.4% (*n* = 50)
Other gender identities	–	11.4% (*n* = 8)
Prefer not to say	0.3% (*n* = 1)	–
Ethnicity		
White British	94.0% (*n* = 296)	84.3% (*n* = 59)
Other White background	3.2% (*n* = 10)	8.6% (*n* = 6)
Mixed/multi-ethnic	1.9% (*n* = 6)	4.3% (*n* = 3)
Asian/British Asian	1.0% (*n* = 3)	1.4% (*n* = 1)
Black/African/Caribbean/Black British	–	1.4% (*n* = 1)
Country of study		
Scotland	87.3% (*n* = 275)	61.4% (*n* = 43)
England	10.5% (*n* = 33)	37.1% (*n* = 26)
Northern Ireland	1.3% (*n* = 4)	1.4% (*n* = 1)
Wales	0.3% (*n* = 1)	–
Mental health conditions^*^		
Anxiety/social anxiety	55.9% (*n* = 176)	77.1% (*n* = 54)
Depression/bipolar	47.6% (*n* = 150)	61.4% (*n* = 43)
Eating disorder	13.7% (*n* = 43)	20.0% (*n* = 14)
Post-Traumatic Stress Disorder	12.1% (*n* = 38)	25.7% (*n* = 18)
Obsessive Compulsive Disorder	6.7% (*n* = 21)	18.6% (*n* = 13)
Personality disorder	6.0% (*n* = 19)	14.3% (*n* = 10)
Substance abuse/Addiction disorder	3.5% (*n* = 11)	18.6% (*n* = 13)
Schizophrenia	0.3% (*n* = 1)	5.7% (*n* = 4)
Other	2.9% (*n* = 9)	2.9% (*n* = 2)
Prefer not to say	1.0% (*n* = 3)	1.4% (*n* = 1)

* *Participants could select more than one mental health condition*.

We also asked participants to self-report mental health conditions. For non-autistic participants, 37.5% (*n* = 118) reported that they had a diagnosed mental health condition, 35.6% (*n* = 112) reported mental health difficulties but no formal diagnosis, and 27% (*n* = 85) reported no mental health conditions. For autistic participants, 68.6% (*n* = 48) had diagnosed mental health conditions, 22.9% (*n* = 16) had suspected mental health conditions, and 8.6% (*n* = 6) had no mental health conditions. Specific self-reported mental health conditions are shown in Table 1, with anxiety and depression the most common conditions for both groups.

Ethical approval for this research was obtained *via* the University of Stirling Ethics Delegated Authority. The questionnaire was also reviewed by an autistic person with lived experience of dropping out of university. They provided feedback on the aims of the study and reviewed the questionnaire in full to ensure that it was accessible and respectful. We also discussed the findings and our interpretations of the study with this individual. Due to funding constraints, deeper participatory involvement was unfortunately not possible, as this study was conducted as part of an undergraduate dissertation. Feedback, however, indicated that this topic was likely important to the autistic community, that the survey was accessible, and that our interpretations were aligned with the data and their personal experiences, with additional insights provided on the effects of the pandemic for autistic people.

### Materials and Procedure

Participants completed an online questionnaire, which was developed in the survey software “Qualtrics.” Participants first completed demographic questions including gender, age, and ethnicity, followed by questions about their degree, such as topic and year of study. We next asked questions regarding whether they had an autism diagnosis (or self-identified) and/or mental health conditions. Participants were also asked whether they believed their mental health had deteriorated since the start of their degree (options: yes/no/prefer not to say). Participants then completed the following measures in the order presented below.

#### Mental Health

The presence of mental health symptoms was examined using the DASS-21 (Lovibond and Lovibond, 1995). There were 21-items related to the symptoms of depression, anxiety, and stress, rated on a 4-point Likert scale [from “did not apply to me at all” (0) to “applied to me very much/most of the time” (3)]. Examples items included statements, such as “I felt that life was meaningless.” Answers mapped onto three subscales, with seven items each corresponding to symptoms of depression, anxiety, and stress. Following DASS-21 procedures, the total score for each subscale was calculated by summing the seven items and multiplying by two, with a range of possible scores from 0 to 42, with higher scores indicating higher depressive, anxiety, or stress symptoms. All subscales had very good internal reliability within each group [Stress: Cronbach’s *α* = 0.83 (non-autistic), *α* = 0.80 (autistic); anxiety: *α* = 0.87 (non-autistic), *α* = 0.85 (autistic); depression: *α* = 0.87 (non-autistic), and *α* = 0.90 (autistic)]. The DASS-21 has previously been validated for use with autistic adults, indicating that this measure can be used to assess depression, stress, and anxiety with this group (Park et al., 2020).

#### Burnout

We used the Copenhagen Burnout Inventory (CBI; Kristensen et al., 2005) to measure burnout. Participants rated statements based on how much the statements applied to them over the past year on a 4-point scale, coded in terms of percentages [“Never (0%), Occasionally (25%), Half of the time (50%), most of the time (75%). or all of the time (100%)”]. We used two subscales from the CBI to look at personal burnout (six items) and work-related burnout, which we modified to focus on academic burnout (seven items). For example, items, such as “Is your work emotionally exhausting,” were modified to “Is your university work emotionally exhausting.” For this study, the subscale of “Client-related burnout” within the CBI was not used as it was deemed irrelevant to the focus of the research. The CBI has been adapted to be used with students before [Campos et al., 2013 (Portugal/Brazil), Sveinsdóttir et al., 2021 (Iceland, nursing students), and Zarobkiewicz et al., 2018 (Poland, medical students)]. For both personal and academic burnout, an average percentage score was calculated, which could range from 0 to 100%, with higher scores indicating a higher degree of burnout. Internal reliability was very good for both personal burnout (non-autistic *α* = 0.85, autistic *α* = 0.86) and academic burnout (non-autistic *α* = 0.88, autistic *α* = 0.89). To the best of our knowledge, this measure has not been used with autistic people before.

#### Coping Styles

We used the Brief-COPE (Carver, 1997) to measure coping styles. The Brief-COPE consists of 28 items where participants rate how often they use different coping techniques on a 4-point Likert scale [from “I haven’t been doing this at all” (1) to “I’ve been doing this a lot” (4)]. For example, “I’ve been getting emotional support from others, such as peers, friends, family, or professionals.” In this study, some question wording was adapted to refer specifically to a university environment (e.g., “I’ve been taking actions to try and make my situation *at university* better”). The original measure can be coded into scores for 14 different types of coping, however, we organized scoring into adaptive (16 items) and maladaptive (12 items) coping styles (Kasi et al., 2012; Choi et al., 2015). Total scores could therefore range from 16 to 64 for adaptive coping and 12 to 48 for maladaptive coping, with higher scores indicating higher use of that coping style. Internal reliability was good for adaptive coping (non-autistic *α* = 0.78, autistic *α* = 0.81) and acceptable for maladaptive coping (non-autistic *α* = 0.67, autistic *α* = 0.68). This measure has previously been validated for use with autistic people (Muniandy et al., 2021).

#### Autistic Characteristics

Autistic characteristics were measured using the Ritvo Autism Asperger Diagnostic Scale (RAADS-14; Eriksson et al., 2013). The RAADS-14 has 14 items, rated on a 4-point Likert scale [from “This was never true and never described me” (0) to “describes me now and when I was young” (3)]. For example, “Some ordinary textures that do not bother others feel offensive when they touch my skin.” Scores could range from 0 to 42, with higher scores indicating greater autistic traits, and a cut-off score above 14 relating to increased likelihood of being autistic. Internal reliability was good (non-autistic *α* = 0.80, autistic *α* = 0.75). A systematic review of screening tools for autism indicated that the RAADS-14 has satisfactory psychometric properties (Baghdadli et al., 2017).

#### Qualitative Questions

Participants were asked “Have you ever considered dropping out of university? And what is the reason for your answer?” Using answers to this question, participants were coded as either considering dropping out or not. Conventional content analysis was used to analyze the reasons why participants had considered dropping out (Hsieh and Shannon, 2005; see below). We also asked: “In what ways do you feel that the Coronavirus pandemic has affected you most (personally and university-wise)?” Answers to this question were also analyzed using conventional content analysis.

#### Design and Data Analysis

We used a cross-sectional mixed methods exploratory survey. Quantitative data were analyzed using SPSS version 27. Data were normally distributed, but due to a significant difference in age between the two groups, age was controlled for in all analyses where possible. For our significance threshold, we considered values of *p* < 0.005 as significant, and values between 0.05 and 0.005 as suggestively significant (Ioannidis, 2018). To examine the first research question (differences in dropout, burnout, coping, and mental health), we used chi-square to examine considering dropping out, due to categorical data, and multivariate analysis of covariance (MANCOVA) for all other measures. We used conventional content analysis (Hsieh and Shannon, 2005, see below) to examine the qualitative reasons given for considering dropping out. To examine the second research question (what predicts considering dropping out), two separate binary logistic regressions were used for each group, with considering dropout (yes/no) as the outcome and age, RAADS score, stress, anxiety, depression, adaptive coping, maladaptive coping, personal burnout, and academic burnout as predictors. For the third research question (what predicts burnout), four separate linear regressions were conducted, looking at personal and academic burnout in each group, with the same predictors as above (controlling for each type of burnout in the analyses). The Variance Inflation Factors (VIF) were all below 5, indicating no multicollinearity. Finally, for the fourth research question (COVID-19), qualitative responses were analyzed using conventional content analysis. For all content analyses, all responses were read through several times by two researchers to gain familiarity with the data. Initial codes were identified and discussed between the two individuals, to compare thoughts on the common experiences present in the data. Codes were then refined and organized into categories, naming the categories based on the commonalities expressed in the data. All responses were then categorized accordingly by one researcher.

## Results

### Descriptive Statistics

When asked if they believed their mental health had deteriorated since the start of their degree, 68.3% (*n* = 215) of non-autistic students said “yes,” 30.5% (*n* = 96) said “no” and 1.3% (*n* = 4) preferred not to say. For autistic students, 75.7% (*n* = 53) said “yes,” 20.0% (*n* = 14) said “no” and 4.3% (*n* = 3) preferred not to say. Means and standard deviations (*SD*) for all measures are shown in Table 2.

**Table 2:** Descriptive statistics (mean, *SD*) for each measure for autistic and non-autistic participants.

	Non-autistic mean (*SD*)	Autistic mean (*SD*)
Stress	21.82 (8.61)	26.29 (7.66)
Anxiety	18.40 (9.85)	22.43 (9.19)
Depression	18.63 (9.48)	23.69 (10.12)
Adaptive coping styles	34.71 (7.12)	35.61 (7.65)
Maladaptive coping styles	27.29 (5.28)	27.94 (5.45)
Personal burnout	54.95 (21.03)	63.04 (20.58)
Academic burnout	58.82 (22.35)	65.71 (23.03)
Autistic characteristics	11.09 (8.47)	30.73 (7.47)

### Dropping Out

For the non-autistic group, 268 participants answered the question about whether they had considered dropping out – 165 (61.6%) indicated that they had, and 103 (38.4%) had not. For the autistic group, 63 participants answered the question, of which 49 (77.8%) had considered dropping out and 14 had not (22.3%). Chi-square indicated that there was a suggestively significant association between group and considering dropping out, *χ*^2^(1) = 5.87, *p* = 0.019, 2-sided, indicating that autistic students were more likely to have thought about dropping out.

Additionally, 49 autistic and 164 non-autistic participants provided an explanation for why they had thought about dropping out. The reasons given were largely very similar for both groups (Table 3). For both, the most frequently cited reason concerned their *mental well-being*, whereby participants explained how university was having a negative mental impact, including increasing stress, anxiety, and depression. The next most frequent reason centered around *doubting it all*, with participants expressing either self-doubt that they could complete their course, doubts that their chosen course was right for them, or it had not met their expectations. Next, most often participants talked about *academic challenges* in terms of aspects of studying, such as high workloads, deadlines, assessments, or failing modules. Other reasons given, reported within less than 10% of responses for both groups, included *social challenges* (such as difficulties making friends or feeling like they did not fit in), *lack of support* (e.g., from their university support services or from lecturers), *financial reasons* (such as feeling university was becoming financially unviable), and finally reasons related to *COVID-19* (such as the switch to online teaching making studying more challenging).

**Table 3:** Number and percentage of responses coded, and example quotes for participants’ reasons for considering dropping out.

Category	Autistic group *n* (% of responses)	Autistic group example quote	Non-autistic group *n* (% of responses)	Non-autistic example quote
Mental well-being	23 (32.9%)	“I was struggling a lot with low mood”	74 (33.5%)	“It’s just seemed like more stress than it’s worth at times”
Doubting it all	17 (24.3%)	“I sometimes feel that maybe I’m just not cut out for it and that I should just accept that”	62 (28.1%)	“I’ve often felt like I wasn’t smart enough to complete my degree”
Academic challenges	11 (15.7%)	“I struggle with the workload, and can’t keep up”	44 (19.9%)	“Having multiple deadlines at the same time and not knowing what to prioritise”
Social challenges	7 (10.0%)	“Friendships forming around me and being completely left out for 4 years”	8 (3.62%)	“I was very lonely due to having made no friends”
Lack of support	6 (8.57%)	“I realised in first year that I was not going to receive any really meaningful support from university services and have felt a bit disillusioned since”	13 (5.88%)	“Yes - due to feeling as though I was not getting the support I needed personally when I needed it the most”
Financial reasons	5 (7.14%)	“Yes, out of concern that the financial investment is not worth it”	8 (3.62%)	“Cost of everything (rent, food, bills) being expensive”
COVID-19	1 (1.46%)	“I have been very stressed due to COVID-19”	12 (5.43%)	“I have considered dropping out and reapplying once full-time face to face teaching resumes”

*N reflects the number of responses coded, and percentages are the percentage out of total responses. Percentages do not add up to 100 as responses could be coded in multiple categories*.

### Mental Health

**Figure 1:**
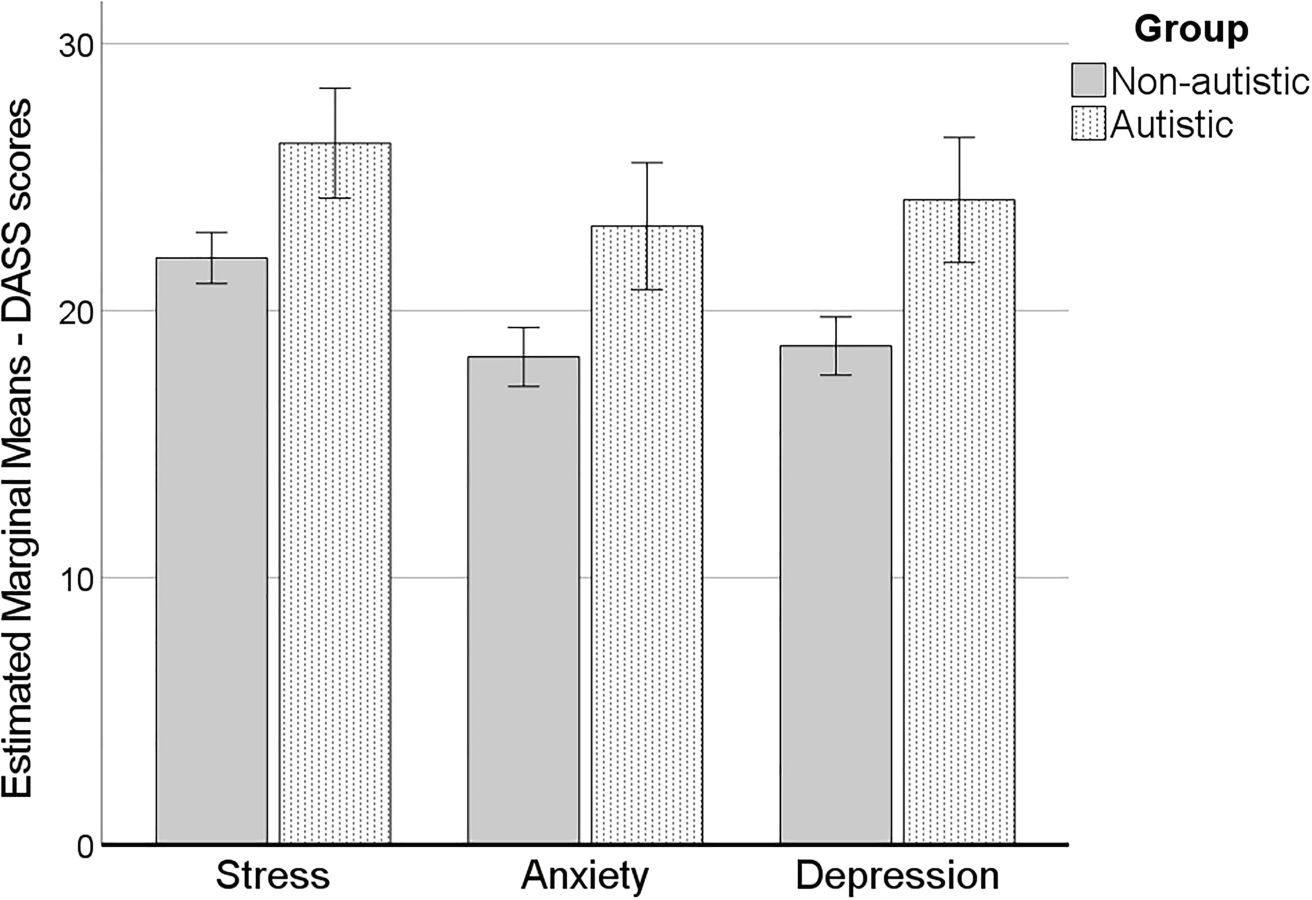
Stress, anxiety, and depression for autistic and non-autistic participants. Errors bars ±2 standard error. Estimated marginal means, controlling for age.

Using Pillai’s Trace, there was a significant main effect of group on mental health, *V* = 0.055, *F*(3, 376) = 7.25, *p* < 0.001. The covariate of age was also significant, *V* = 0.050, *F*(3, 376) = 6.60, *p* < 0.001. Subsequent univariate ANCOVAs showed there was a significant difference for stress [*F*(1, 378) = 14.04, *p* < 0.001, $\eta_{p}^{2}$ = 0.036], anxiety [*F*(1, 378) = 13.73, *p* < 0.001, $\eta_{p}^{2}$ = 0.035] and depression [*F*(1, 378) = 17.64, *p* < 0.001. $\eta_{p}^{2}$ = 0.045], such that autistic participants experienced higher rates of each of these (Figure 1). The covariate age only suggestively significantly related to anxiety [*F*(1, 378) = 7.69, *p* = 0.006, $\eta_{p}^{2}$ = 0.020], such that anxiety decreased with age.

### Coping

For coping styles, Pillai’s trace indicated that there was no main effect of group, *V* = 0.005, *F*(2, 343) = 0.83, *p* = 0.44, $\eta_{p}^{2}$ = 0.005, indicating no difference in coping styles between the groups. The covariate, age, was also not significant [*V* = 0.009, *F*(2, 343) = 1.47, *p* = 0.23, $\eta_{p}^{2}$ = 0.009].

### Burnout

**Figure 2:**
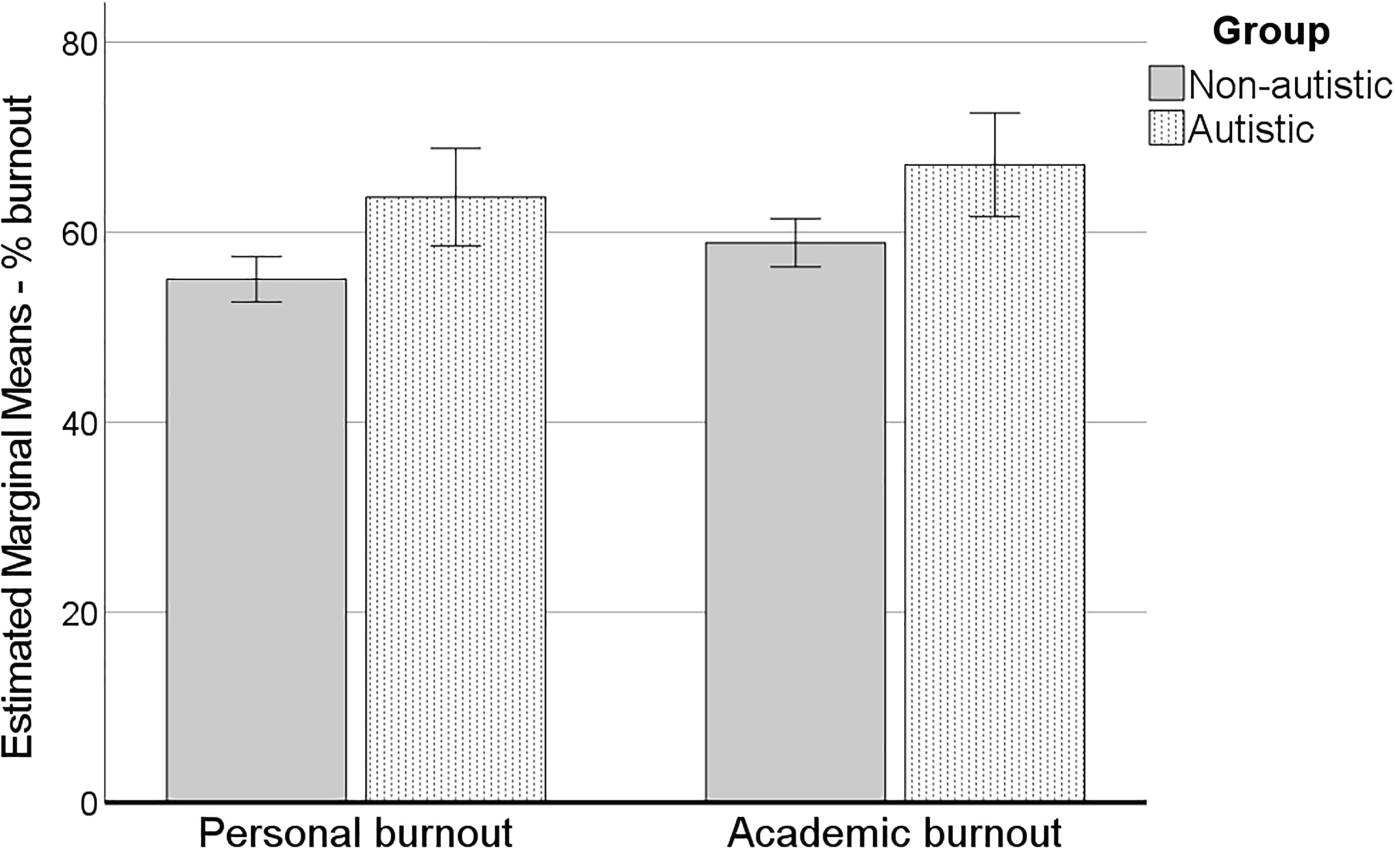
Personal and academic burnout for autistic and non-autistic participants. Errors bars ±2 standard error. Estimated marginal means, controlling for age.

For burnout, Pillai’s trace indicated a suggestively significant main effect of group, *V =* 0.024, *F*(2, 377) = 4.73, *p* = 0.009. There was also a suggestively significant main effect of the covariate age, *V* = 0.016, *F*(2, 377) = 3.06, *p* = 0.048. Follow-up univariate ANCOVAs indicated there was a significant difference between groups both for personal burnout [*F*(1, 378) = 9.19, *p* = 0.003, $\eta_{p}^{2}$ = 0.024] and a suggestively significant difference for academic burnout [*F*(1, 378) = 7.29, *p* = 0.007, $\eta_{p}^{2}$ = 0.019], such that both types of burnout were higher for the autistic participants (Figure 2). Age was only suggestively significant for academic burnout [*F*(1, 378) = 5.19, *p* = 0.023, $\eta_{p}^{2}$ = 0.014], such that younger age related to higher academic burnout.

### Predicting Considering Dropout

Binary logistic regression was used to examine predictors of considering dropping out. For autistic participants (*n* = 62), the model was significantly better at predicting the outcome than using the constant alone, *χ*^2^(9) = 25.96, *p* = 0.002. Overall, the model correctly classified 83.9% of participants. However, no individual predictors significantly predicted considering dropping out (Table 4).

**Table 4:** Logistic regression results for autistic and non-autistic groups, analyzing predictors of considering dropping out.

	Wald statistic	B (SE)	*p*	Exp(B)	C.I Exp (B)
*Autistic group (n = 62)*					
Age	0.31	0.041 (0.074)	0.58	1.04	[0.90–1.21]
RAADS score	1.07	0.061 (0.059)	0.301	1.06	[0.95–1.19]
Stress	1.25	−0.13 (0.11)	0.26	0.88	[0.71–1.10]
Anxiety	0.037	−0.013 (0.069)	0.85	0.99	[0.86–1.13]
Depression	2.09	0.088 (0.061)	0.15	1.09	[0.97–1.23]
Adaptive coping	1.68	−0.098 (0.075)	0.20	0.91	[0.78–1.05]
Maladaptive coping	1.45	0.18 (0.15)	0.23	1.20	[0.89–1.61]
Personal burnout	2.99	0.076 (0.044)	0.084	1.08	[0.99–1.18]
Academic burnout	0.000	0.000 (0.031)	0.99	1.00	[0.94–1.06]
*Non-autistic group (n = 265)*					
Age	1.096	0.047 (0.045)	0.30	1.05	[0.96–1.14]
RAADS score	1.93	0.029 (0.021)	0.16	1.03	[0.99–1.07]
Stress	0.066	−0.008 (0.031)	0.80	0.99	[0.93–1.05]
Anxiety	0.350	0.012 (0.021)	0.55	1.01	[0.97–1.06]
Depression	0.43	0.013 (0.021)	0.51	1.01	[0.97–1.06]
Adaptive coping	0.002	−0.001 (0.022)	0.97	0.99	[0.96–1.04]
Maladaptive coping	6.88	0.095 (0.036)	0.009^**^	1.10	[1.02–1.18]
Personal burnout	0.078	0.004 (0.013)	0.78	1.004	[0.98–1.03]
Academic burnout	5.81	0.027 (0.011)	0.016^**^	1.03	[1.01–1.05]

*B (SE) = unstandardized beta coefficient, standard error; Exp (B) = odds ratio; C.I Exp (B) = 95% confidence intervals for odds ratio. Autistic group Cox and Snell R square = 0.34, Nagelkerke R square = 0.53; non-autistic group Cox and Snell R square = 0.22, Nagelkerke R square = 0.30*.

** *p = 0.005–0.05 considered suggestively significant*.

For non-autistic participants (*n* = 265), the model was significantly better at predicting the outcome than using the constant alone, *χ*^2^(9) = 65.37, *p* < 0.001. Overall, the model correctly classified 73.2% of participants. Greater use of maladaptive coping styles and higher academic burnout related to significantly increased likelihood of considering dropping out, at a suggestively significant threshold (Table 4).

### Predicting Burnout

Considering academic burnout, for autistic participants, the model explained 65.7% of the variance and was significant [*F*(8, 62) = 15.81, *p* < 0.001]. However, the only significant predictor of academic burnout was personal burnout (Table 5). For non-autistic participants, the model explained 65.8% of the variance and was significant [*F*(8, 273) = 66.74, *p* < 0.001]. Here, greater use of maladaptive coping styles, higher personal burnout, and younger age predicted academic burnout. At a suggestively significant threshold, higher stress and lower anxiety moderately predicted academic burnout (Table 5) – these findings are treated with caution.

**Table 5:** Linear regression results for autistic and non-autistic groups, analyzing predictors of academic burnout.

	B	B CI	SE B	*β*	*p*	*f* ^2^
*Autistic group (n = 63)*						
Age	−0.087	[−0.65–0.48]	0.28	−0.025	0.76	0.0005
Autistic characteristics	0.039	[−0.47–0.55]	0.25	0.012	0.88	0.0001
Stress	0.39	[−0.44–1.23]	0.41	0.13	0.35	0.005
Anxiety	−0.31	[−0.89–0.26]	0.29	−0.12	0.28	0.007
Depression	0.28	[−0.22–0.77]	0.25	0.12	0.26	0.007
Adaptive coping	−0.38	[−0.87–0.11]	0.25	−0.13	0.13	0.013
Maladaptive coping	0.38	[−0.47–1.24]	0.43	0.090	0.37	0.005
Personal burnout	0.74	[0.47–1.02]	0.14	0.66	<0.001^*^	0.20
*Non-autistic group (n = 274)*						
Age	−0.68	[−1.13–-0.24]	0.23	−0.11	0.003^*^	0.012
Autistic characteristics	−0.14	[−0.34–0.06]	0.10	−0.053	0.17	0.002
Stress	0.35	[0.035–0.67]	0.16	0.13	0.030^**^	0.006
Anxiety	−0.22	[−0.44–0.00]	0.11	−0.097	0.050^**^	0.005
Depression	0.11	[−0.11–0.33]	0.11	0.047	0.33	0.001
Adaptive coping	−0.072	[−0.31–0.17]	0.12	−0.023	0.55	0.0004
Maladaptive coping	0.93	[0.56–1.30]	0.19	0.22	<0.001^*^	0.031
Personal burnout	0.67	[0.55–0.78]	0.056	0.63	<0.001^*^	0.21

*B unstandardized beta coefficient, B CI confidence intervals at 95% lower and upper bounds, SE B standard error, β standardized beta coefficient, f^2^ effect size (0.02 considered small effect, 0.15 medium, 0.35 large)*.

* *p < 0.005 considered significant,*

** *p = 0.005–0.05 considered suggestively significant*.

For personal burnout, the model explained 71.9% of the variance and was significant [*F*(8, 62) = 20.83, *p* < 0.001] for autistic participants. However, as before, the only significant predictor of personal burnout was academic burnout (Table 6). For non-autistic participants, the model explained 70.2% of the variance and was also significant [*F*(8, 273) = 81.24, *p* < 0.001]. Higher anxiety, stress, autistic characteristics, and academic burnout all significantly predicted greater personal burnout (Table 6).

**Table 6:** Linear regression results for autistic and non-autistic groups, analyzing predictors of personal burnout.

	B	B CI	SE B	*β*	*p*	*f* ^2^
*Autistic group (n = 63)*						
Age	0.18	[−0.28–0.63]	0.23	0.055	0.44	0.003
Autistic characteristics	0.26	[−0.15–0.66]	0.20	0.091	0.21	0.007
Stress	0.46	[−0.21–1.12]	0.33	0.17	0.18	0.009
Anxiety	0.32	[−0.14–0.79]	0.23	0.14	0.17	0.009
Depression	0.051	[−0.35–0.45]	0.20	0.026	0.80	0.0003
Adaptive coping	0.15	[−0.26–0.55]	0.20	0.055	0.47	0.002
Maladaptive coping	0.44	[−0.24–1.13]	0.34	0.12	0.20	0.008
Academic burnout	0.49	[0.31–0.66]	0.088	0.54	<0.001^*^	0.16
*Non-autistic group (n = 274)*						
Age	0.15	[−0.25–0.55]	0.20	0.026	0.45	0.0006
Autistic characteristics	0.23	[0.058–0.41]	0.089	0.093	0.009^**^	0.007
Stress	0.55	[0.28–0.82]	0.14	0.22	<0.001^*^	0.017
Anxiety	0.29	[0.095–0.49]	0.099	0.13	0.004^*^	0.009
Depression	0.19	[−0.009–0.39]	0.10	0.085	0.061	0.004
Adaptive coping	−0.030	[−0.24–0.18]	0.11	−0.010	0.78	0.00008
Maladaptive coping	−0.19	[−0.53–0.15]	0.17	−0.047	0.28	0.001
Academic burnout	0.52	[0.44–0.61]	0.044	0.55	<0.001^*^	0.18

* *p < 0.005 considered significant,*

** *p = 0.005–0.05 considered suggestively significant*.

### How Has the COVID-19 Pandemic Affected Autistic and Non-autistic Students?

In total, 62 autistic and 267 non-autistic participants provided responses to our question about how COVID-19 had affected them. Using content analysis, we identified several similarities in the experiences of autistic and non-autistic students during the pandemic (Table 7). Most frequently, both groups talked about how the pandemic had led to *social isolation and limited social opportunities.* This category reflected how participants felt socially isolated, missed their friends and family, and felt they were unable to connect with other students on their courses. Next, most often both groups discussed how *online university is harder, overwhelming and unmotivating*. Here, many participants felt the emergency shift to online teaching had made their courses much more difficult and stressful, that they often found it hard to stay motivated and engaged, and ultimately to complete their academic work. The third most cited category for both groups centered on the *negative impact on mental and physical well-being*, with participants talking about how aspects of both mental and physical well-being had become worse during the pandemic, such as increased anxiety and depression. For non-autistic participants, the fourth most mentioned category was *trapped within these same four walls*, whereby they described how they felt physically isolated and hemmed in by having to work and study in the same place. Autistic participants mentioned this category less often, instead, their fourth most cited category was *a lack or loss of support*, where they described the ways the pandemic had resulted in support either being reduced, or not enough support being put in place, particularly from university services. Some of the non-autistic participants also mentioned this category as an issue.

**Table 7:** Number and percentage of responses coded, and example quotes for participants’ responses when asked about how COVID-19 had affected them.

Category	Autistic group *n* (%)	Autistic example quote	Non-autistic group *n* (%)	Non-autistic example quote
Social isolation and limited social opportunities	34 (27.0%)	“I do miss the social aspects of going to uni physically”	126 (26.3%)	“Isolated from friends and family, made it more difficult to meet peers on course/build friendships”
Online university is harder, overwhelming and unmotivating	29 (23.0%)	“Online lectures make it hard to build up motivation to attend and complete personal study”	118 (24.6%)	“I am really struggling to engage and keep up with online learning and my deadlines are making me much more stressed than usual”
Negative impact on mental and physical well-being	16 (12.7%)	“Escalated anxieties and declined mental health significantly”	59 (12.3%)	“Every day is a struggle to keep going, I have to fight to make it each day. Good thing I’m pretty tough with mental health crises at this point”
A lack or loss of support	15 (11.9%)	“The university is not making any efforts to provide accessible teaching or well-being support for students”	34 (7.10%)	“You aren’t getting the same support from peers/lecturers online as you would face to face and in person”
Online university is good for me	9 (7.14%)	“Improved situation at university because I work better in my own comfortable surroundings, much easier to get work done rather than becoming distracted/anxious at university”	22 (4.59%)	“It has actually helped me to attend more classes by being able to do them from home”
Increased uncertainty and worries about the future	8 (6.35%)	“I have also struggled to cope with the uncertainty and transition to employment”	18 (3.76%)	“I feel bombarded with bad things happening in the world and realistically how much does my degree that doesn’t have a career attached matter.”
Positive opportunities for me personally	7 (5.56%)	“I’ve had more time for me through the summer and been able to manage my interactions with others far more”	25 (5.22%)	“Been able to focus on myself and get myself fit and healthy again and learn how to control emotions”
COVID-specific fears and worries	6 (4.76%)	“I am finding it almost impossible to complete work during the pandemic because I have the extra stress of worrying about the pandemic”	14 (2.92%)	“There’s the fear of doing something wrong without knowing or passing on an illness you didn’t know you had.”
Trapped within these same four walls	2 (1.59%)	“Can’t really go out and feel isolated”	56 (11.7%)	“Studying, eating, sleeping and chilling all in the same place is difficult - there is no escape from the environment you study.”
Negative financial impact	0	n/a	7 (1.46%)	“It has changed my financial situation drastically which has made living costs very difficult to cover”

*N reflects the number of responses coded, and percentages are the percentage out of total responses. Percentages do not add up to 100 as responses could be coded in multiple categories*.

Less than 10% of responses were also coded into other categories, with participants in both groups occasionally talking about *increased uncertainty and worries about the future*, describing how they had struggled with all the uncertainty and changes brought about by the pandemic, and were concerned about how things would pan out in the future, often in terms of their career. Additionally, some participants in both groups talked about *COVID-specific fears and worries*, with the pandemic itself, its associated restrictions, regulations, and the risks of catching or spreading the virus to others, being of significant concern. Only non-autistic students mentioned the *negative financial impact* that the pandemic had had on them. Finally, in both groups some participants noted positive effects – some explained how *online university is good for me*, whereby they found that the changes to teaching were more accessible or enabling for them. Others also talked about *positive opportunities for me personally*, reflecting on how the pandemic had given them time and space to focus on or learn more about themselves.

## Discussion

This study aimed to explore autistic and non-autistic students’ experiences in relation to dropout, burnout, mental health, coping, within the context of the COVID-19 pandemic and a challenging time for Higher Education. We found that autistic students were more likely to have thought about dropping out and reported higher rates of burnout, anxiety, stress, and depression. However, we did not identify any significant predictors of considering dropping out for autistic students, but greater use of maladaptive coping strategies and higher academic burnout predicted non-autistic students considering dropping out. Looking at the phenomenon of burnout, only the two types of burnout measured predicted one another for autistic students, while there were several predictors for non-autistic students including age, maladaptive coping strategies, autistic traits, stress, and anxiety. From qualitative responses, it was clear that the COVID-19 pandemic had a significant impact on the social and emotional lives of both autistic and non-autistic participants, with many challenges associated with emergency online learning. Altogether, our findings indicate several important implications and avenues for further research.

Given the suggestion that autistic students are more likely to drop out of university, our study adds some further practical and theoretical insight on this topic. Concerningly high numbers of both autistic and non-autistic students reported they had thought about dropping out, with autistic students more likely to report thinking about this (77.8% autistic versus 61.6% non-autistic). Looking at why autistic students may be more likely to consider dropping out, in our quantitative analyses, we did not find any significant predictors for our autistic participants. However, our predictors focused on individual, psychological variables, rather than sociological ones (Behr et al., 2020). We did not measure aspects such as academic skills, transition experiences or fitting in at university, or poor autism acceptance, which past research has indicated may link to dropping out for autistic students (Cage et al., 2020; Cage and Howes, 2020). Further theoretical work is needed which considers the role of the wider ecosystem around the student when it comes to dropping out, as has been outlined for autistic students graduating from university (Vincent and Fabri, 2020).

Some further practical insight can be drawn from our qualitative analysis of the reasons for thinking about dropping out, where interestingly there was little difference between the reasons given by autistic and non-autistic students. Instead, the most frequently cited reasons for both groups were difficulties related to poor mental well-being, doubting themselves and/or their course, and academic challenges. These qualitative findings support calls for universities to promote positive well-being *via* high-quality support services and trained staff (Hill et al., 2020), who know how to support *both* autistic students and those with mental health difficulties and to develop students’ self-efficacy and self-confidence *via* supportive networks throughout universities (Wilcox et al., 2005). Further qualitative work would be beneficial to further explore autistic students’ thoughts about dropping out in-depth, as well as more comprehensive quantitative work to model student dropout, taking into account both individual and societal variables (Behr et al., 2020).

Burnout is a variable which has been little explored in relation to autistic students’ experiences at university, despite burnout being a theoretically important variable in autistic peoples’ well-being (Higgins et al., 2021). Although burnout did not predict thoughts about dropping out for the autistic participants, overall burnout was significantly higher for this group. As there is limited research on burnout for autistic people, our study adds further evidence concerning this phenomenon among autistic students and we suggest this should be an area of high priority for further research. Looking at predictors of burnout, for autistic students, we found only each type of burnout (personal and academic) predicted each another. This finding implies that each form of burnout feeds into one another and captures an overall construct of burnout – if someone is generally exhausted, they also feel exhausted with academic life, and vice versa. Additionally, since we could identify predictors of burnout for the non-autistic group but not the autistic group, this suggests there is theoretical validity in considering a specific experience of autistic burnout (Raymaker et al., 2020; Higgins et al., 2021). Unmeasured variables unique to autistic burnout may play a greater role than those measured in the current study. Further, among our non-autistic students, we found that greater autistic characteristics predicted personal burnout. The fact that autistic characteristics predicted burnout may indicate that those with autistic traits may invest energy into masking these or experience some stigma associated with their traits, linking to depleted mental resources and more burnout. More work is clearly needed in this area, including validating measures of burnout (academic, personal, and autistic) and understanding practical ways of mitigating the effects of burnout.

In addition to high burnout levels, we also found that autistic participants reported higher rates of depression, anxiety, and stress, which has important practical implications. This finding fits with the extant literature on autistic mental health more generally, which shows higher prevalence (e.g., Lai et al., 2019), and corroborates Gurbuz et al.’s (2019) findings which indicated higher mental health difficulties for autistic students, but did not use validated measures of symptoms. Higher quality mental health support for autistic students is clearly needed, ideally designed *with* and *for* autistic students, rather than simply adapting non-autistic supports (Gunin et al., 2021). Our findings on coping styles could also provide insight on how autistic students could be supported to cope with mental health difficulties. Interestingly, we found no difference in coping styles between autistic and non-autistic students, which could indicate that there are few differences in the ways these students cope with stress. Alternatively, it could be that we missed some of the different coping strategies used by autistic people (Muniandy et al., 2021) – in an interview study, Anderson et al. (2020) identified “working hard,” “part-time enrolment,” “extended breaks,” “changing discipline,” and “camouflaging” as strategies former autistic students had used to cope at university. Given the high levels of mental health challenges, burnout, and thoughts about dropping out, further research on the coping strategies of autistic students would be useful so that we can better understand how these students can be best supported to cope with the pressures of university life.

In this study, we were also interested in the experiences of our participants within the context of the COVID-19 pandemic, which also provides some important implications for universities as we continue to navigate this pandemic at the time of writing. We looked at experiences qualitatively, noting many similarities between our autistic and non-autistic participants. For all, the most reported challenge related to the social impact of the pandemic – over a quarter of both groups described how social disconnection had negatively affected them. This finding mirrors Pellicano et al.’s (2021) research with Australian autistic people and goes against the stereotyped view that autistic people lack social interest and our findings support counter-arguments to the theoretical proposal that autistic people lack social motivation (Jaswal and Akhtar, 2018). University can offer many social opportunities for autistic people, and while they may face challenges within social environments (Scott and Sedgewick, 2021), we must find ways to enable autistic students to flourish socially in pandemic-adapted universities. Particularly, making the social environment fit for the autistic student, rather than the other way round, is vital (Vincent et al., 2017). For example, societies, clubs, and university events (e.g., “Welcome Week”) should consider how they can be accessible to autistic students. This accessibility might be achieved by providing clear information in advance about social events, making sure events are in well-designed sensory spaces or using peer mentoring programs (Cage et al., 2020; Scott and Sedgewick, 2021). Indeed, peer support programs for autistic students have shown promise (Duerksen et al., 2021). Considering the high rates of considering dropping out, burnout, and mental health difficulties noted in this study – including how both groups qualitatively reported the negative impact the pandemic had had on their mental well-being – social support could be a crucial factor (Mostert and Pienaar, 2020), which needs further investigation.

Additionally, around a quarter of participants in both groups mentioned not being satisfied with the provision of emergency online teaching at their university. Our participants commonly reported finding academic work harder, less motivating, and overwhelming. These findings support other research with autistic and non-autistic students during the pandemic (Aristovnik et al., 2020; De Man et al., 2021; Gin et al., 2021; Monahan et al., 2021). In the United Kingdom, the shift to emergency online teaching happened quickly, and studies of United Kingdom academic staff indicated that many viewed the shift as negative, detrimental both to staff well-being and academia itself (Watermeyer et al., 2021). Indeed, a survey of students in Switzerland indicated that the difficulties lecturers had in adapting suddenly to online teaching was contributing to stress for the students (Lischer et al., 2021). In our study, a few participants (in both groups) reported that they found online learning was a positive experience for them, and their experience may depend on how the participants’ respective universities shifted their teaching online. It is important to bear in mind that universities had to switch to online teaching rapidly, and therefore the emergency online provision is not equivalent to a true “flipped classroom” model, which would effectively utilize asynchronous online learning combined with synchronous “in class” engagement. In theory, online learning *should* be more accessible, and this “flipped classroom” should promote more active learning, help students to engage with material, and encourage greater collaboration between students (Flores et al., 2016). Moving forward, academic staff should consider guidelines on how to effectively teach online, and the opportunities that shifting online could offer in the long term (Nordmann et al., 2020).

Finally, our findings from our non-autistic participants also have additional implications. For these students, considering dropping out was predicted by higher rates of maladaptive coping and academic burnout. Past research has found similar relationships between coping, burnout, and dropping out, with maladaptive coping strategies linking to burnout, and then burnout having a knock-on effect on dropout intention (Marôco et al., 2020). Marôco et al. (2020) suggest actions to promote student engagement and reduce burnout, such as by reducing the volume of assessments, increasing social support, and considering guided interventions. Interestingly, we found that academic burnout was also predicted by more maladaptive coping for non-autistic participants. This finding has been noted elsewhere (Vizoso et al., 2019), and these coping strategies may not present the individual with solutions to the stress they are experiencing, but exacerbate it, thus increasing burnout (Alarcon et al., 2011). Maladaptive coping styles can feed into poor mental health, so interventions focused on promoting and guiding adaptive coping styles may be useful (Tran and Lumley, 2019). University support services could consider offering such interventions to potentially help mitigate dropout and reduce burnout.

### Limitations and Future Directions

This study is limited by a non-generalizable sample, with the views of female, White, Scottish students contributing to the majority of the data. Our sample of autistic students was also relatively small and underpowered (*n* = 70), but larger than previous studies which have directly compared autistic and non-autistic students (e.g., Gurbuz et al., 2019). Due to this small sample size, we were not able to explore the relationships with demographic or student variables, such as whether there were differences between genders, ethnicities, country of study, level of study, or year. Further, many of our autistic students were self-identifying rather than formally diagnosed. However, all self-identifying participants scored highly on the measure of autistic traits, and people who self-identify are often subject to stigma and disbelief, particularly if they do not fit the stereotypic view of “autism” (Lewis, 2017; Leedham et al., 2020). Of note is that the majority of our self-identifying autistic participants were female, and they are likely to experience barriers to accessing diagnosis based on gendered assumptions about autistic people (Leedham et al., 2020). It is important too to consider how not having or struggling to access a diagnosis could impact support and university experiences. Indeed, in interviews with autistic people who dropped out of university, many explained how they had not received their diagnosis until after university, but wished that they had known earlier (Cage and Howes, 2020). Further research could examine the experiences of those who self-identify in more detail to examine whether this may be a group at particular risk of dropping out.

We also do not have pre-pandemic data, and all findings must be considered within the context of the pandemic. For example, without pre-pandemic data, it is difficult to assess whether mental well-being had worsened for either group – although other United Kingdom studies with non-autistic students suggest this has happened (Savage et al., 2020), and research with autistic people (non-students) suggest mental health worsened dependent on COVID-related distress (Adams et al., 2021). Longitudinal, follow-up data would be useful in monitoring how students’ experiences pan out. Additionally, we only considered a small set of variables, and have undoubtedly missed critical contextual and nuanced analysis, including being able to examine interactions between variables due to the small sample size for autistic students. Nonetheless, our findings add to the limited comparative literature on autistic students’ experiences and highlight the continued need to improve the quality of support provided.

Additionally, we found much higher thoughts about dropping out compared to previous studies, for example, Gurbuz et al. (2019), who found 56% autistic students (total *n* = 26) had considered dropping out compared to only 15.3% of non-autistic students (total *n* = 158) – compared to 77.8% of autistic and 61.6% non-autistic in our slightly larger sample. There may be several explanations for our higher rate which reflect limitations in our study: our sample is not representative, and the survey may have particularly attracted non-autistic students with mental health conditions who wished to share their experiences, and who may be more likely to think about dropping out, in part due to systemic failures of universities in supporting students with mental health difficulties (O’Keeffe, 2013). Further, we broadly asked whether our participants had “ever thought about dropping out.” Many students may have *thought* about dropping out but may not act on those thoughts. Our study thus may not have accurately captured the intent. Nonetheless, our study supports findings that indicate autistic students are more likely to drop out of university (e.g., Newman et al., 2011), and a *thought* about dropping out could easily build to eventually deciding to withdraw from university. Finally, our analysis of predictors of dropping out is underpowered due to the small number of autistic students who said that they had *not* thought about dropping out, and this under-powering may have contributed to us being unable to identify specific predictors. Despite these limitations, we believe it is important not to invalidate our participants’ experiences, especially given the challenges they qualitatively described in terms of their mental well-being.

### Conclusion

Our study highlights how our autistic participants were more likely to have thought about dropping out, alongside higher burnout and greater anxiety, depression, and stress. Most often, autistic students mentioned low mental well-being as the reason they were thinking about dropping out. Future work should focus on promoting positive well-being, and actions taken to do this could alleviate some of the other issues mentioned in this study. For example, accessible, high-quality support services, training academic staff (particularly about mental health, autism, and effective online teaching strategies), and reviewing how learning and teaching can support rather than burnout students, could all help create universities where well-being is prioritized. Additionally, our findings related to COVID-19 show the value of social aspects of university, for *all* students. Opportunities to connect with other students must be designed with (neuro)diversity in mind – for example, having social events in calm sensory environments, focusing on shared interests and passions, or developing peer mentoring or “buddying” schemes. As we look toward the future, universities must concentrate on creating inclusive, accessible, and supportive environments.

## Data Availability Statement

The raw data supporting the conclusions of this article will be made available by the authors, on reasonable request to the corresponding author.

## Ethics Statement

The studies involving human participants were reviewed and approved by University of Stirling General University Ethics Panel Delegated Authority. The patients/participants provided their written informed consent to participate in this study.

## Author Contributions

EM conceived of the study, collected the data, and contributed to subsequent drafts. EC helped to design the project, conducted the data analyses, and wrote the draft of the manuscript. All authors approved the final version.

## Funding

Open access publication fees were supported by the University of Stirling APC fund. Some additional financial support was provided by the Department of Psychology at the University of Stirling.

## Conflict of Interest

The authors declare that the research was conducted in the absence of any commercial or financial relationships that could be construed as a potential conflict of interest.

## Publisher’s Note

All claims expressed in this article are solely those of the authors and do not necessarily represent those of their affiliated organizations, or those of the publisher, the editors and the reviewers. Any product that may be evaluated in this article, or claim that may be made by its manufacturer, is not guaranteed or endorsed by the publisher.

## Footnotes

^1^Identity first language is used throughout this paper to respect the preferences of the autistic community (Kenny et al., 2016; Bury et al., 2020) and avoid ableist language (Bottema-Beutel et al., 2020).
